# Risk Factors for Kala-Azar in Bangladesh

**DOI:** 10.3201/eid1105.040718

**Published:** 2005-05

**Authors:** Caryn Bern, Allen W. Hightower, Rajib Chowdhury, Mustakim Ali, Josef Amann, Yukiko Wagatsuma, Rashidul Haque, Katie Kurkjian, Louise E. Vaz, Moarrita Begum, Tangin Akter, Catherine B. Cetre-Sossah, Indu B. Ahluwalia, Ellen Dotson, W. Evan Secor, Robert F. Breiman, James H. Maguire

**Affiliations:** * Centers for Disease Control and Prevention, Atlanta, Georgia, USA;; † International Centre for Diarrhoeal Disease Research, Bangladesh, Dhaka, Bangladesh

**Keywords:** disease outbreaks, Epidemiology, leishmaniasis, risk factors, Visceral, prevention & control, Bangladesh/epidemiology

## Abstract

Since 1990, South Asia has experienced a resurgence of kala-azar (visceral leishmaniasis). To determine risk factors for kala-azar, we performed cross-sectional surveys over a 3-year period in a Bangladeshi community. By history, active case detection, and serologic screening, 155 of 2,356 residents had kala-azar with onset from 2000 to 2003. Risk was highest for persons 3–45 years of age, and no significant difference by sex was seen. In age-adjusted multivariable models, 3 factors were identified: proximity to a previous kala-azar patient (odds ratio [OR] 25.4, 95% confidence interval [CI] 15–44 within household; OR 3.2 95% CI 1.7–6.1 within 50 m), bed net use in summer (OR 0.7, 95% CI 0.53–0.93), and cattle per 1,000 m^2^ (OR 0.8, 95% CI 0.70–0.94]). No difference was seen by income, education, or occupation; land ownership or other assets; housing materials and condition; or keeping goats or chickens inside bedrooms. Our data confirm strong clustering and suggest that insecticide-treated nets could be effective in preventing kala-azar.

Since 1990, South Asia has experienced a resurgence of the lethal parasitic disease visceral leishmaniasis (VL). India, Bangladesh, and Nepal account for an estimated 300,000 cases annually and 60% of the global burden (in terms of disability-adjusted life years lost) of VL (1,2). Superimposed on this poorly controlled VL-endemic situation are outbreaks that affect hundreds of thousands of people, as in Bihar in the early 1990s (3). The full-blown clinical syndrome caused by VL is characterized by fever, weight loss, splenomegaly, hepatomegaly, skin darkening, and anemia and is known as kala-azar ("black fever" in Hindi). Kala-azar is nearly always fatal if untreated (4). Even with treatment, case-fatality rates often exceed 10% in VL-endemic areas of Asia and Africa (5).

*Leishmania donovani* is transmitted by the female sand fly, and humans are the only reservoir in South Asia (6). Blanket residual insecticide spraying decreased the incidence of kala-azar below detectable levels in India and Bangladesh by the 1960s (3), which suggests that sustained vector control could substantially reduce disease prevalence today. Efforts to control this neglected disease have recently gained momentum from the government of India's commitment to eliminate kala-azar by the year 2010 (7). Nevertheless, data on the epidemiology of anthroponotic VL are sparse. To plan effective strategies for VL control and elimination, we must understand patterns of disease occurrence both at the community level and at broader geographic and ecologic levels. To elucidate the determinants at the community level, we studied spatial patterns and risk factors for kala-azar in a highly affected community in Bangladesh.

## Methods

The study design was based on cross-sectional household surveys from January to April in 2002, 2003, and 2004. The surveys included leishmaniasis serologic studies and active kala-azar case detection. The study physician (M.A.) was present during the surveys and at regular intervals between surveys and offered free diagnosis for residents with suspected kala-azar; thus, additional ascertainment occurred between surveys. The protocol was approved by the International Centre for Diarrhoeal Disease Research, Bangladesh (ICDDR,B) Research and Ethical Review Committees and the Institutional Review Board of the Centers for Disease Control and Prevention (CDC). Informed consent was obtained from all adult participants and a parent or guardian of all participating children. Assent was also obtained from children ≥7 years of age.

The study community is located in Fulbaria Thana, Mymensingh District, the "thana" (subdistrict) that has consistently reported the highest kala-azar incidence in Bangladesh since 2000. Community members identified the seasons as winter (October 15–March 15), summer (March 15–June 15), and rainy season (June 15–October 15). The community houses ≈12,000 people and is divided into 9 "paras" (sections) of ≈100 to 500 households. The paras are separated by 1 to 2 km and physically are much like separate villages, but politically they are considered parts of the same community. The study area comprised the 3 paras with the highest kala-azar rates during the previous several years according to villagers' reports; these were designated paras 1–3 (8). The study included all members living in the study area for >6 months in the 3 years before the 2002 survey. Household questionnaires were used to collect data on births, deaths, inward and outward migration, socioeconomic factors, animal ownership, and house construction. Individual questionnaires focused on sleeping location, bed net use, and dietary practices.

### Kala-Azar Case Ascertainment

During the 2002 survey, we attempted to retrospectively identify all kala-azar cases that had occurred in the study population. From January 2002 onward, ascertainment was prospective. Suspected kala-azar cases were identified through structured interviews by trained fieldworkers and referred to the study physician for complete medical history and physical examination. All seropositive persons were also examined. We defined a past case of kala-azar as an illness with >2 weeks of fever that resolved after 20 days of intramuscular injections and included a history of 1 or more of the following symptoms: weight loss, abdominal fullness, abdominal pain, or skin darkening. Patients who died with a disease consistent with kala-azar were also included; several patients died during treatment. While most patients could not identify the specific drug used, the 20-day course of injections corresponds to the sodium stibogluconate (SSG) regimen prescribed as first-line treatment by the national kala-azar management guidelines. We defined current kala-azar cases based on history and physical examination (symptoms as for past cases, plus splenomegaly or hepatomegaly, with or without measured fever or jaundice) and positive serologic test results. To confirm suspected kala-azar, we used the rK39 enzyme-linked immunosorbent assay (ELISA) during serosurveys and rK39 dipstick between serosurveys. The rK39 dipstick (Inbios International, Seattle, WA, USA) is a rapid test for kala-azar with very high sensitivity and moderately high specificity (9,10).

All persons with active kala-azar were referred to the Thana Health Complex located ≈1 km away. Patients with an atypical presentation or suspected relapse were referred to the district hospital for bone marrow aspiration and parasitologic confirmation. The study provided generic SSG (GlaxoWellcome-Bangladesh); each batch was tested by the International Dispensary Association (Amsterdam, the Netherlands) to ensure pharmacologic quality. Because of the ongoing SSG shortage in Bangladesh (8), the study provided SSG for all new kala-azar patients in the community, whether or not the patient lived in a study para.

### Serologic Methods

Capillary blood specimens were collected from consenting participants ≥3 years of age. The serologic assay used recombinant K39 antigen (Corixa Corporation, Seattle WA, USA). In 2002, we used rK39 ELISA methods based on published protocols in which human antibodies reacting with plate-bound *L. donovani* antigens were detected with horseradish-conjugated protein A (11). The positive cutoff was initially based on the mean optical density (OD) value of 4 wells of pooled negative control sera plus 3 standard deviations as described previously (11). Substantial plate-to-plate variation was seen in the negative control mean, and standard deviations were small; positive cutoff OD values were often close to the negative control mean. Therefore, to improve specificity for active kala-azar case confirmation, we used an alternative cutoff of 10 standard deviations (strong seropositive) for 2002 serosurvey data. We subsequently refined our methods to address these issues. For 2003 and 2004 surveys, we included a standard curve of dilutions of a pool of known positive sera and based our cutoff on concentration units from the standard curve for each plate (manuscript in preparation). We also used horseradish peroxidase–conjugated goat anti-human immunoglobulin (Ig) G, IgA, and IgM (Biosource International, Camarillo, CA, USA) because this reagent yielded better specificity than protein A conjugate. The negative cutoff was established by using serum specimens from persons from areas of Bangladesh not endemic for VL. Based on this assessment, we defined an ELISA reading of >60 concentration units as strongly seropositive.

### Analytic Methods

All study households were mapped by Global Positioning System, and data were uploaded into ArcView Geographic Information System (GIS), v.3.3 (ESRI, Redlands, CA, USA). By using GIS data, distance was calculated from each household to the closest kala-azar case in the preceding year, and for multivariable modeling, to the closest case in any of the preceding years. To evaluate the effect of cattle (cows, oxen, or calves) on kala-azar risk for nearby residents, kernel density estimation was used to estimate cattle per 1,000 m^2^. This index provides a smoothed measure of both proximity and number of cattle. Corresponding to where cattle were kept at night, the cattle shed was considered the center of gravity. If the household had no shed, house location was used based on the common practice of keeping cattle close to the house to prevent theft. A 50-m cell size was used to weight the analysis toward cattle in close proximity to a household, regardless of ownership.

Data were analyzed by using SAS 8.02 (SAS Institute Inc, Cary, NC, USA). Univariate and multivariable models were adjusted for within-household correlation by using Generalized Estimating Equations. Multivariable models were constructed by stepwise addition of variables significant at the p = 0.05 level in univariate analyses.

## Results

A total of 2,439 persons in 506 households met the inclusion criteria. The surveyed population was 48% male and 52% female; median age was 18 years (range 0–80). Among those 20–29 years of age, 40% were male and 60% female; some adult men worked in Dhaka or Mymensingh and did not meet the inclusion criteria. For other age groups, the sex distribution was similar. The median household size was 5 persons (range 1–11).

During the 2002 serosurvey, 148 study participants had a history of kala-azar treatment, and active kala-azar was diagnosed in 16 patients. From the end of the 2002 survey through April 2004, active kala-azar was diagnosed in 49 additional participants, for a total of 65 prospectively ascertained kala-azar cases. In addition, probable relapses were diagnosed in 3 previously treated kala-azar patients; 6 previously treated patients were diagnosed with post–kala-azar dermal leishmaniasis. Altogether, we were able to reliably assign status with respect to kala-azar for 2,356 (97%) of 2,439 persons, of whom 213 (9%) had kala-azar or a history of kala-azar, 58 with onset before 2000 and 155 with onset in 2000 or later. Subsequent analyses focused on the 155 kala-azar patients with onset in 2000 or later because these data were considered the most reliable and complete.

The ELISA results were strongly positive for 33 (97%) of 34 prospectively ascertained kala-azar patients tested; the remaining patient had an ELISA reading of 57, just below the cutoff of 60 concentration units. The other 31 prospectively ascertained kala-azar cases were confirmed by rK39 dipstick (27 patients) or bone marrow aspirate (4 patients). Treated kala-azar patients also had strongly positive serologic test results that persisted for years after clinical recovery; 13 (24%) of 54 patients treated in 2001 and 13 (50%) of 26 patients treated in 2002 remained seropositive in 2004. Because so few patients had definitive parasitologic diagnosis, a formal analysis of serology performance characteristics was not performed.

**Figure 1:**
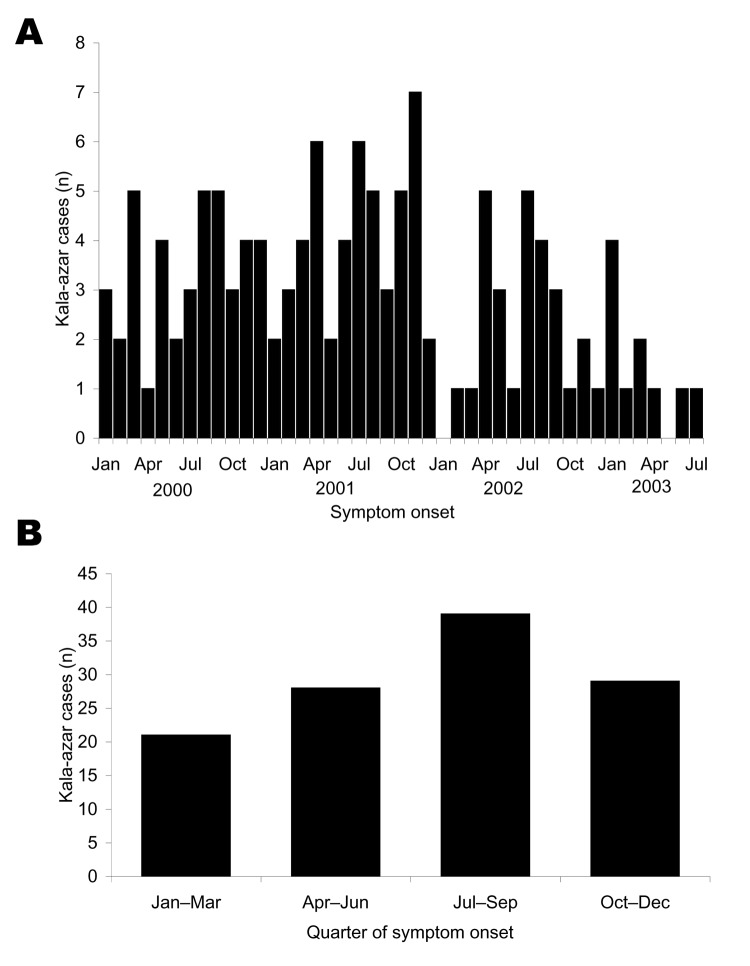
A) Kala-azar cases by symptom-onset month, Bangladesh, January 2000 to August 2003. B) Kala-azar cases by quarter of symptom onset, based on aggregated data, 2000–2002. Ascertainment for cases with onset in 2003 was not complete at the time of analysis.

Para 1 had a higher cumulative incidence (77 [14%] of 540) than para 2 (39 [3%] of 1,221) or para 3 (39 [7%] of 537, p<0.01 for all three 2-way comparisons). Kala-azar incidence peaked in 2001: 47 residents had illness onset in 2000, 57 in 2001, 23 in 2002, and 28 in 2003. No marked seasonal pattern was apparent by onset month (Figure 1A). Because ascertainment of cases with onset in 2003 was incomplete at the time of analysis, we combined quarterly data from January 2000 to December 2002 (Figure 1B). The quarterly analysis showed a trend for more cases to have onset from July to September and fewer cases from January to March (goodness-of-fit χ^2^ = 5.63, p = 0.13). The incidence was slightly higher in men than women (p = 0.27) and was higher among children and young adults than in the youngest and oldest age groups (p<0.01) (Table 1). The median symptom duration before treatment was 4.0 months. Among patients treated before the study began, the duration was longer for female patients (5.0 months) than male patients (3.0 months, p = 0.09). After we began active case finding, this trend disappeared (3.5 and 4.0 months for female and male patients, respectively, p = 0.82). The case-fatality rate was 9% (14/155), 14% among female patients and 5% among male patients (relative risk 2.7, p = 0.07).

**Table 1:** Cumulative incidence of kala-azar (KA) from January 2000 to December 2003 in a Bangladeshi community*

Age (y)	Male		Female		All	
	n	KA patients (%)	n	KA patients (%)	n	KA patients (%)
<3	113	3 (2.7)	103	1 (1.0)	216	4 (1.9)
3–14	396	40 (10.1)	425	31 (7.3)	821	71 (8.7)
15–45	462	33 (7.1)	540	41 (7.6)	1,002	74 (7.4)
>45	127	5 (3.9)	123	1 (0.8)	250	6 (2.4)
All	1,098	81 (7.4)	1,191	74 (6.2)	2,289	155 (6.8)

*Patients with onset before 2000 (n = 58) were excluded.

**Figure 2:**
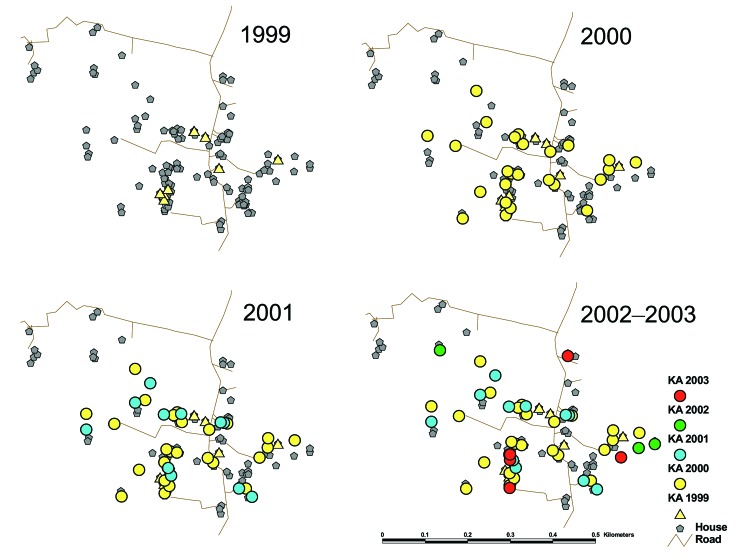
Location of kala-azar patients by year of symptom onset in para 1, Bangladesh, 2000–2003.

From 2000 to 2003, kala-azar cases spread from a highly clustered pattern to one in which substantial sections of the study village were saturated (Figure 2). Kala-azar risk was significantly higher among those living in the same household as or within 50 m of a kala-azar patient in the previous year (p = 0.0003 for closest patient in 1999 as predictor of kala-azar in 2000, p<0.0001 for closest patient in 2000 as predictor in 2001). By 2002, the pattern had disseminated so that the difference in incidence based on proximity was no longer significant in the overall study population (p = 0.12). In 2000, 21% of the study population lived within 50 m of a patient in the previous year; this figure rose to 37% in 2001 and 53% in 2002. When cumulative incidence during the study period was considered, 72% of the population lived within 50 m of a kala-azar case by 2003; in para 1, the proportion was 84% (Figure 2).

A number of other factors were associated with altered kala-azar risk (Table 2). The risk of kala-azar was highest for people in the 3- to 14-year and 15- to 45-year age groups. Consistent use of a bed net, especially in summer, was strongly protective (Table 2). The nets in use were not treated with insecticide and were locally produced. Other factors related to bed nets, such as net use in winter, household net ownership, and having ≥1 net per 3 household members, were associated with weaker levels of protection (Tables 2 and 3). Overall, 91% of households owned at least 1 net, and 87% of participants reported sleeping under a net at least some of the time.

**Table 2:** Individual-level factors associated with kala-azar (KA) in univariate analyses adjusted for household clustering, Bangladesh, 2000–2003

Factor	N*	Cumulative KA incidence, n (%)	OR (95% CI)†	p value
Age (y)				
<3	216	4 (1.9)	0.78 (0.22–2.78)	0.71
3–14	821	71 (8.7)	3.99 (1.77–8.99)	0.0008
15–45	1,002	74 (7.4)	3.28 (1.48–7.27)	0.0034
>45	250	6 (3.9)	Referent	
Female	1,196	74 (6.2)	0.84 (0.62–1.15)	0.29
Male	1,102	81 (7.4)	Referent	
Sleeps on ground	150	9 (6.0)	0.99 (0.51–1.90)	0.97
Sleeps on bed or cot	1,849	123 (6.7)	Referent	
Always uses net in summer	1,769	96 (5.4)	0.43 (0.29–0.64)	<0.0001
Uses net sometimes or never in summer	370	46 (12.4)	Referent	
Always uses net in winter	754	30 (4.0)	0.49 (0.33–0.73)	0.0004
Uses net sometimes or never in winter	1,385	112 (8.1)	Referent	
Always uses net in rainy season	1,277	76 (6.0)	0.79 (0.54–1.17)	0.24
Uses net sometimes or never in rainy season	862	66 (7.7)	Referent	
Uses bed net ever	1,849	109 (5.9)	0.51 (0.32–0.80)	0.0033
Never uses bed net	290	33 (11.4)	Referent	
Distance from previous KA patient				
Same house	468	72 (15.4)	35.7 (21.1–60.2)	<0.0001
<50 m	1,178	67 (5.7)	3.51 (1.89–6.51)	<0.0001
>50 m	652	16 (2.5)	Referent	
Eats beef at least twice a month	711	49 (6.9)	1.1 (0.76–1.59)	0.62
Eats beef less than once a month	1,299	83 (6.4)	Referent	
Eats goat at least twice a month	206	9 (4.4)	0.59 (0.24, 1.43)	0.24
Eats goat less than once a month	1,804	123 (6.8)	Referent	
Eats fish daily	1,402	96 (6.9)	1.25 (0.80–1.94)	0.33
Eats fish less than daily	610	36 (5.9)	Referent	
Eats chicken at least twice a month	437	34 (7.8)	1.34 (0.86–2.11)	0.2
Eats chicken less than once a month	1,567	98 (6.3)	Referent	
Goats kept in sleeping room	248	14 (5.7)	0.87 (0.48–1.57)	0.64
No goats in sleeping room	1,705	108 (6.3)	Referent	
Chickens kept in sleeping room	1,269	80 (6.3)	1.04 (0.68–1.60)	0.85
No chickens in sleeping room	681	42 (6.2)	Referent	

*Existing values for each variable. †OR, odds ratio; CI, confidence interval.

We examined the effect of both cattle ownership and cattle density on kala-azar risk. Household cattle ownership was associated with lower risk, but this finding did not reach significance (p = 0.18, Table 3). However, the kernel density analysis demonstrated a significant protective effect for increasing cattle density: the mean density around the houses of persons without kala-azar was 1.4 cows/1,000 m^2^ compared to 1.1 cows/1,000 m^2^ for kala-azar patients (odds ratio 0.75, 95% confidence interval 0.62–0.92, p = 0.005). A dose-response relationship was seen; with 0 cows/1,000 m^2^ as the referent, ≤1 cow/1,000 m^2^ was associated with a 30% decrease, 1.1–2 cows/1,000 m^2^ with 40% decrease, and >2 cows/1,000 m^2^ with 43% decrease in kala-azar risk.

**Table 3:** Household-level factors associated with kala-azar (KA) in univariate analyses adjusted for household clustering, Bangladesh, 2000–2003

Household characteristic	N*	Cumulative KA incidence, n (%)	OR (95% CI)†	p value
Farmer or farm laborer	1,459	99 (6.8)	0.99 (0.78–1.27)	0.97
Other occupation	753	49 (6.5)	Referent	
Monthly income ≥$10 per person	1,096	67 (6.1)	0.82 (0.55–1.22)	0.33
Monthly income <$10 per person	1,112	81 (7.3)	Referent	
Owns ≥0.2 acres of land	1,186	80 (6.8)	1.06 (0.72–1.58)	0.76
Owns <0.2 acres of land	1,026	68 (6.6)	Referent	
Owns a bed net	2,012	125 (6.2)	0.58 (0.35–0.96)	0.033
Does not own a bed net	198	21 (10.6)	Referent	
≤3 household members per net	924	41 (4.4)	0.53 (0.35–0.79)	0.0023
>3 household members per net	1,374	114 (8.3)	Referent	
Owns bicycle	186	10 (5.4)	0.71 (0.36–1.42)	0.33
Does not own a bicycle	2,026	138 (6.8)	Referent	
Owns radio	497	24 (4.8)	0.65 (0.39–1.08)	0.1
Does not own a radio	1,715	124 (7.2)	Referent	
Head of household can read	652	48 (7.4)	1.2 (0.78–1.83)	0.41
Head of household cannot read	1,560	100 (6.4)	Referent	
Keeps cattle	1,050	67(6.4)	0.89 (0.75–1.06)	0.18
Keeps no cattle	912	63 (6.9)	Referent	
Roof made of tin	1,441	93 (6.5)	1.12 (0.70–1.79)	0.64
Roof made of thatch or mixed	511	32 (6.3)	Referent	
Earthen walls	1,689	113 (6.7)	1.81 (0.77–4.21)	0.17
Bamboo, tin, or concrete walls	269	12 (4.5)	Referent	
Damp floor	1,607	103 (6.4)	1.17 (0.59–2.31)	0.65
Dry floor	346	19 (5.5)	Referent	

*Existing values for each variable. †OR, odds ratio; CI, confidence interval.

No difference was seen in kala-azar risk by income, education, or occupation; assets such as land, livestock, farm implements, radio, or cart; housing materials and condition; dietary intake of selected foods; keeping livestock inside human sleeping rooms; or history of residual insecticide spraying in the last 5 years (Tables 2 and 3).

In the final multivariable model, 4 factors remained significant. Age from 3 to 45 years and proximity to a previous patient increased kala-azar risk, while higher numbers of cattle per 1,000 m^2^ and consistent use of a bed net in summer were associated with protection (Table 4).

**Table 4:** Multivariable model of factors associated with kala-azar (KA), adjusted for household clustering

Factor	OR (95% CI)*	p value
Always uses net in summer	0.69 (0.52–0.92)	0.01
Uses net sometimes or never	Referent	
Distance from previous KA case		
Within household	25.6 (15.0–43.7)	<0.0001
<50 m	2.9 (1.6–5.4)	0.0006
>50 m	Referent	
Each cow per 1,000 m^2^	0.81 (0.70–0.94)	0.005
No cattle	Referent	
Age (y)		
<3	0.7 (0.2–2.0)	0.46
3–14	3.6 (1.7–7.5)	0.0008
15–45	3.8 (1.9–7.8)	0.0002
>45	Referent	

*OR, odds ratio; CI, confidence interval.

## Discussion

This study is the first to examine spatial patterns and risk factors for anthroponotic VL in Bangladesh. Although we were not surprised that proximity to previous cases was a determinant of subsequent kala-azar risk, the strength of the association was remarkable. The 26-fold increase in risk for those living with a patient reflects the role of active kala-azar patients as the predominant infection reservoir. In this study, the mean duration of illness before treatment was 4 months; this delay provides ample opportunity for sand flies that feed inside the house to become infected and transmit disease. The risk associated with kala-azar patients within 50 m but not farther may reflect the relatively limited flight range of sand flies.

To present data applicable to regional control efforts, we chose to focus our analysis on risk factors for kala-azar, the form of VL reported in surveillance data and the predominant target of public health programs. In this analysis, we did not distinguish between uninfected participants and those with possible subclinical leishmanial infection. For this reason, we may have underestimated the strength of association for factors that alter risk of both leishmanial infection and disease by including infected persons in our control group. At the same time, factors that may alter the risk of progression from infection to disease were not examined. For example, immunogenetic factors play a role in determining whether people infected with *Leishmania infantum* (*chagasi*) progress to clinical disease (12). Similar genetic factors are likely to affect progression of *L. donovani* and may contribute to the household clustering in our data. Poor nutritional status may also alter risk of progression (13). Although our analysis showed no significant risk variation with socioeconomic or dietary indicators, poor nutritional status may vary by household resources and practices, contributing to the high risk for members of kala-azar households.

Previous studies in South Asia demonstrated associations between kala-azar and poverty (14,15). However, the facility-based survey in India and case-control study in Nepal compared kala-azar patients to the general population, whereas in the current analysis we studied a relatively homogeneous, high-risk population. Our data showed no differences in risk by occupation, income, housing type, or assets aside from bed nets. A community-based study of kala-azar in India demonstrated an association with agricultural occupation, but like our study, no association between kala-azar and household income levels (16). The primary occupation in the Indian village was weaving; <10% of villagers were farmers. In contrast, 66% of our population lived in agricultural households. The lack of significance of socioeconomic factors may reflect the relative homogeneity of our study population and the fact that at the community level, more proximate factors determine kala-azar risk.

Our findings with respect to cattle answer a question raised by the Nepal case-control study (15). In that study, owning cattle or buffaloes conferred a strong protective effect. However, because of the study design, it was impossible to distinguish among 3 potential explanations for this effect: socioeconomic confounding, better nutritional status leading to decreased progression to kala-azar, and the role of bovines as a preferred sand fly bloodmeal source. The finding that cattle ownership was not as important as cattle density strongly suggests that cows decrease leishmaniasis transmission by sand flies in their immediate vicinity. Sand fly bloodmeal analysis in India confirms that *Phlebotomus argentipes* feed predominantly on bovines, with humans as their second choice (17). The proximity of cattle may diminish disease transmission by enabling sand flies to feed preferentially on animals not susceptible to leishmaniasis, thereby decreasing sand fly parasite acquisition, feeding on humans, or both.

Remarkably, untreated, locally available bed nets were associated with a 30% decrease in kala-azar risk in our multivariable model. Because the usual incubation period for kala-azar is 2–6 months (18), the strong protective effect of net use in March–June and the higher kala-azar incidence in the third quarter of the year are consistent with high transmission in the Bangladeshi summer. Nevertheless, some transmission probably occurs year-round, except for December–January when almost no sand flies are active. In addition, the extremes of the incubation period are highly variable, with a reported range from 10 days to >2 years (18,19), making strict seasonal correlation difficult.

The protective effect of untreated nets in this analysis is consistent with findings of the Nepal kala-azar case-control study (15). Intervention trials of insecticide-treated materials for anthroponotic cutaneous leishmaniasis in Afghanistan demonstrate strong protective efficacy (20), and the high rate of use in our data suggests that bed nets are already highly acceptable in VL-endemic communities. Indeed, at the community's request, insecticide-treated nets were distributed to the population when the study ended. These findings highlight the promise of insecticide-treated nets as a VL control measure that could be implemented and sustained through community action (8). Our findings suggest that in VL-endemic areas where treated nets are not yet available, untreated nets should be used whenever possible.

The strong spatial clustering we found suggests that targeted vector-control efforts, such as spraying to the most affected foci, could be effective if they are instituted before the transmission pattern generalizes. Furthermore, our data suggest that generalization may occur within 2 to 3 years when transmission is intense. Thus, rapidity of response may be key to the success of a targeted intervention. The several-year delays that commonly occur before spraying in affected communities may help explain the low efficacy of current targeted spraying programs in South Asia. The incompleteness of kala-azar surveillance data may be another factor. Nonetheless, better disease control might be achieved through improved kala-azar surveillance systems that integrate a rapid, targeted, vector-control response mechanism. In addition, combining government-run spraying programs with community-level efforts to increase insecticide-treated net use could enhance vector control.

Maintenance of adequate kala-azar diagnostic and treatment facilities at the peripheral level will also be essential (8). Rapid diagnostic tests such as the rK39 dipstick and the direct agglutination test now make diagnosing most kala-azar cases possible without invasive procedures (21). Two new antileishmanial drugs, miltefosine and paromomycin, are or soon will be available in India, where antimonial drug resistance presents a major challenge to control (22). Both have advantages over currently used antileishmanial drugs, miltefosine because of its oral administration and paromomycin because of its excellent safety profile. The low rate of relapse in our study suggests that most VL in Bangladesh is still responsive to SSG. Nevertheless, if miltefosine and paromomycin can be made available in Bangladesh and Nepal at affordable prices, treatment could be made simpler and more effective. The resurgence of kala-azar in South Asia since 1990 has raised policymakers' awareness of this historically neglected disease, which suggests that the political will may finally exist to address VL in a concerted fashion (23). The time has come to mount an aggressive, integrated effort to control anthroponotic visceral leishmaniasis.
